# Face masking and COVID-19: potential effects of variolation on transmission dynamics

**DOI:** 10.1098/rsif.2021.0781

**Published:** 2022-05-04

**Authors:** Zachary Levine, David J. D. Earn

**Affiliations:** Department of Mathematics and Statistics, McMaster University, Hamilton, Canada L8S 4K1

**Keywords:** infectious diseases, COVID-19, variolation, face masks, SIR model, differential equations

## Abstract

Face masks do not completely prevent transmission of respiratory infections, but masked individuals are likely to inhale fewer infectious particles. If smaller infectious doses tend to yield milder infections, yet ultimately induce similar levels of immunity, then masking could reduce the prevalence of severe disease even if the total number of infections is unaffected. It has been suggested that this effect of masking is analogous to the pre-vaccination practice of variolation for smallpox, whereby susceptible individuals were intentionally infected with small doses of live virus (and often acquired immunity without severe disease). We present a simple epidemiological model in which mask-induced variolation causes milder infections, potentially with lower transmission rate and/or different duration. We derive relationships between the effectiveness of mask-induced variolation and important epidemiological metrics (the basic reproduction number and initial epidemic growth rate, and the peak prevalence, attack rate and equilibrium prevalence of severe infections). We illustrate our results using parameter estimates for the original SARS-CoV-2 wild-type virus, as well as the Alpha, Delta and Omicron variants. Our results suggest that if variolation is a genuine side-effect of masking, then the importance of face masks as a tool for reducing healthcare burdens from COVID-19 may be under-appreciated.

## 1. Introduction

Early in the COVID-19 pandemic, face masking was discouraged outside healthcare settings [1,2], and inadequate supplies of masks created significant challenges for healthcare workers [3–5]. By 3 April 2020 appreciation of the potential value of masking had increased sufficiently for the US Centers for Disease Control and Prevention (CDC) to recommend the wearing of face coverings in public [6]. Similar recommendations were made in Canada on 6 April 2020 [7] and in the UK on 11 May 2020 [8]. Over the course of the pandemic, evidence that masking is an effective tool to reduce community transmission of SARS-CoV-2 has continued to accumulate [9–16].

An additional potential benefit of masking—even in situations where it fails to prevent transmission—was proposed in the summer of 2020 by Gandhi & Rutherford [17] and Gandhi *et al*. [18]. They noted that if transmission occurs in the presence of face masks, then the infecting viral inoculum is likely to be smaller than is typical when masks are not worn. If smaller inocula tend to lead to less severe infections, then—even if masks fail to block transmission completely—masking could reduce morbidity and mortality from COVID-19 and boost the level of herd immunity in the population.

The notion that promoting mild infections could be an effective disease control strategy has a long history. In the eighteenth century, it was common to infect children with smallpox intentionally—a process known as *variolation*—by administering a small inoculum of smallpox virus taken from an infected person [19–24]. While SARS-CoV-2 infections are never intentional, Gandhi & Rutherford [17] refer to SARS-CoV-2 transmission via small inocula that penetrate masks as variolation. In this paper, we explore the potential benefits of SARS-CoV-2 variolation using a mathematical model.

We begin in §2 by reviewing evidence that supports the hypothesis that face masking may promote SARS-CoV-2 variolation. In subsequent sections, we present and analyse a simple model that allows us to investigate potential effects of variolation induced by face masking.

## 2. Variolation as a side effect of face masking

Face masks can reduce the probability of transmission either through outward or inward filtration. Outward filtration occurs when droplets containing viral inoculum are captured when leaving the mouth or nose of an infected person [25]. Inward filtration occurs when viral particles are prevented from entering someone’s nose or mouth.

Gandhi & Rutherford [17] suggested that people who are infected with SARS-CoV-2 when wearing a face mask might experience less severe illness, because the size of the viral inoculum they receive is reduced by inward filtration. Assuming that individuals infected in this way still develop lasting immunity, they can be considered variolated. Variolation in this sense might not reduce the total proportion of the population infected, but would nevertheless benefit the population by increasing the proportion of infections that are mild or asymptomatic [17].

The variolation hypothesis, as originally formulated by Gandhi & Rutherford [17], depends on three key assumptions:

**Reduced inocula**: individuals who are infected while wearing a mask receive a smaller viral inoculum than if they had not been masked.
**Reduced severity**: smaller viral inocula tend to yield infections with milder symptoms, i.e. there is a positive dose–response relationship.
**Acquired immunity**: mild infections still provide long-lasting natural immunity to the disease.

Reduced inocula is plausible because masks are known to filter droplets that may contain virus particles [26].

Reduced severity from smaller inocula is indicated by several lines of evidence. The dose–response relationship is supported by an investigation in Madrid that found that ‘distinct sizes of viral inoculum at the time of exposure’ could explain different illness courses in three clusters of SARS-CoV-2 infection (infecteds in different clusters developed COVID-19 with different severity, and the only clear difference among the clusters was the degree of SARS-CoV-2 exposure) [27]. In addition, disproportionately severe disease outcomes were documented following a choir rehearsal in the state of Washington in March 2020 [28]; the observed severity of illness among attendees is consistent with a positive dose–response relationship because infected individuals likely spew many more virus particles when singing, compared to simply talking at a social gathering. Finally, an experimental study exposed hamsters to SARS-CoV-2 in a laboratory and found that COVID-19 symptoms were much less severe in infected animals that were shielded by a surgical mask partition [29].

Immunity after mild infection is suggested by a 2020 study in the New York City area, where 13.7% of a sample of healthcare personnel (HCP) were found to have antibodies to SARS-CoV-2 [30]; HCP were not asked if they were symptomatic, but 5% (11%) of those who tested positive for SARS-CoV-2 antibodies reported low (medium) likelihood of SARS-CoV-2 exposure. Another study found that 15% of those with SARS-CoV-2 antibodies reported never having COVID-19 symptoms [31]. Other investigations have revealed that antibodies to SARS-CoV-2 were present in individuals five months after asymptomatic or mild infections [32,33]. Thus, asymptomatic and mild SARS-CoV-2 infections likely do provide at least some protection against reinfection.

## 3. Model

We investigate the potential effects of facemask-induced variolation by expanding the standard Susceptible-Infectious-Removed (SIR) model [34,35].

We assume that all members of the population are identical—and that the population is homogeneously mixed—so we do not distinguish between situations in which poor facemasks are worn universally by everyone versus situations in which better masks are worn by a subset of the population; thus, masks are implicitly assumed to be distributed to individuals at random.

We capture the variolating effect of facemasks by imagining that adherence to facemasking causes a proportion m of infections to be mild, and we therefore refer to m as the *probability of mild infection*. Mild infections might be shorter and/or less transmissible (however, the *effective* infectious period for severe infections could, in practice, be shorter than for mild infections, because severe cases are likely to be isolated quickly). Thus, three distinct effects—causing mild infections, reducing transmission rate and potentially shortening infectious periods—contribute to the overall *effectiveness of mask-induced variolation*. We allow for the possibility that immunity decays [36], but assume the duration of immunity is the same following mild or severe infections.

**Figure 1.**
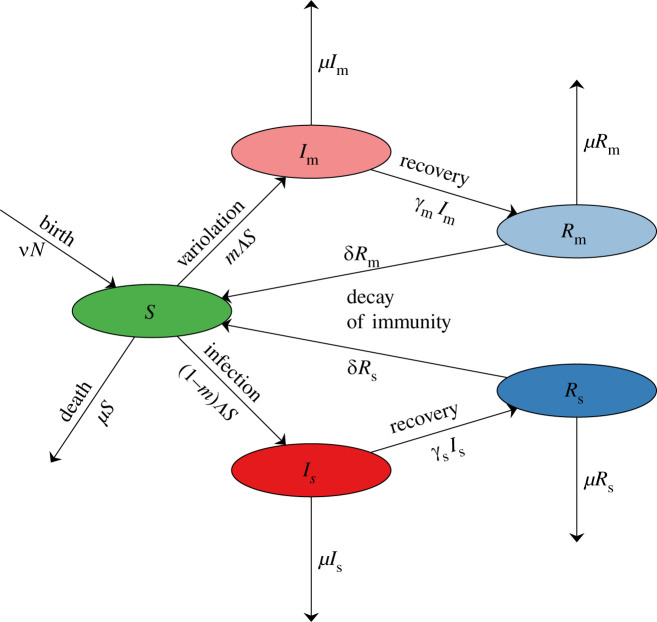
Flow chart for the model defined by equation (3.1). The state variables are defined in table 1, and the parameters are defined in table 3.

Figure 1 presents a flow chart for the model, which we represent formally as a system of ordinary differential equations (ODEs),

3.1a $$\;\frac{dS}{dt}=\nu N-\Lambda S-\mu S+\delta (N-S-I_{m}-I_{s}),$$

3.1b $$\;\frac{dI_{m}}{dt}=m\Lambda S-\gamma_{m}I_{m}-\mu I_{m},$$

3.1c $$\;\frac{dI_{s}}{dt}=(1-m)\Lambda S-\gamma_{s}I_{s}-\mu I_{s},$$

3.1d $$\;\frac{dR_{m}}{dt}=\gamma_{m}I_{m}-\mu R_{m}-\delta R_{m}$$

3.1e $$\mathrm{and}\;\;\;\;\frac{dR_{s}}{dt}=\gamma_{s}I_{s}-\mu R_{s}-\delta R_{s},$$

where the *force of infection* is

3.1f $$\Lambda =\beta_{m}\frac{I_{m}}{N}+\beta_{s}\frac{I_{s}}{N},$$

and the total population size is

3.1g $$N=S+I_{m}+I_{s}+R_{m}+R_{s}.$$

**Table 1.** State variables for the model specified in figure 1 and equation (3.1). All variables represent numbers of individuals.

state variable	meaning
*S*	*susceptible* to infection
$I_{m}$	*mildly infectious* (variolated)
$I_{s}$	*severely infectious*
$R_{m}$	*removed*, after mild infection
$R_{s}$	*removed*, after severe infection

All the state variables are listed in table 1. $R_{m}$ and $R_{s}$ do not appear in the first three lines of equation (3.1) and can be ignored for the purposes of dynamical analysis. We retain equations (3.1*d*,*e*) for convenience, to keep track of the numbers of immune individuals who had mild versus severe infections.

**Figure 2.**
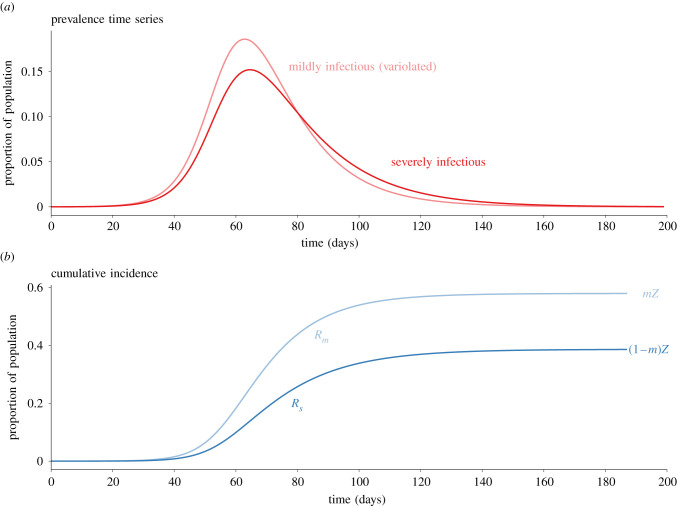
Solution curves for the model (3.1) with the parameter values specified in table 3 (birth rate for Canada). The initial conditions were $\left(S,I_{m},I_{s})/N=(0.9999,0.00008,0.00002\right)$. Panel (*b*) shows the proportions in the removed compartments (approximately cumulative incidence in those compartments), which converge to the indicated values derived in equation (4.18).

Estimated values of parameters associated with COVID-19 are listed in table 2. We use these estimates to set default values for the parameters of our model, which are listed in table 3. Since we ignore the latent stage in our model, we use the estimated mean generation interval for COVID-19 in place of the mean infectious period in our model (cf. [49,52]). We ignore disease-induced mortality, but we (more than) compensate for this by assuming for simplicity that the death rate from other causes (*μ*) is equal to the birth rate (*ν*); thus, births are balanced by deaths and the population size (*N*) is constant. Figure 2 shows prevalence and cumulative incidence time series, obtained by solving equation (3.1) numerically with the default parameters.

**Table 2.** Parameter estimates (and 95% confidence intervals) for COVID-19; these estimates are examples from published studies and are not based on a systematic review of the literature. WT refers to the wild-type virus. The latent period (time from exposure to infectiousness) is extremely difficult to estimate, so the incubation period (time from exposure to appearance of symptoms) is often used as a proxy. The incubation period for the Alpha variant was estimated to be $\frac{2}{3}\times (T_{\mathrm{inc}}\;\mathrm{for}\;\mathrm{WT})$ [37]. $R_{0}$ is expressed in terms of the model parameters in equation (4.1). $R_{0}$ for the Alpha, Delta and Omicron variants were approximated using estimates of the increase in transmissibility for each successive variant, i.e. $\text{WT}\;\overset{1.5\times }{⟶}\;\text{Alpha}$ [38], $\text{Alpha}\;\overset{1.5\times }{⟶}\;\text{Delta}$ [39–41] and $\text{Delta}\;\overset{4.2\times \;[2.1\times -9.1\times ]}{⟶}\;\text{Omicron}$ [42].

parameter	meaning	estimate	references
*T*_lat_	mean latent period	3.7 [3.3–3.9] days	WT [43]
		4.0 [3.5–4.4] days	Delta [44]
*T*_inc_	mean incubation period	6.4 [4.9–8.5] days	WT [45]
		4.2 [3.2–5.6] days	Alpha [37]
		5.8 [5.2–6.4] days	Delta [44]
		∼3 days	Omicron [46]
*T*_inf,m_	mean infectious period for mild infections	9 [6–11] days	[47]
*T*_inf,s_	mean infectious period for severe infections	14 [8–20] days	[48]
*T*_gen,m_	mean generation interval for mild infections	*T*_lat_ + *T*_inf,m_ days	[49, equation (4.1)]
*T*_gen,s_	mean generation interval for severe infections	*T*_lat_ + *T*_inf,s_ days	[49, equation (4.1)]
*T*_imm_	mean immune period	7 [6–8] months	[32,33,50]
$R_{0}$	basic reproduction number	3 [2.1–4.6]	WT [38]
		4.5 [3.15–6.9]	Alpha [51]
		6.75 [4.7–10.4]	Delta [39–41]
		28.4 [9.9–94.6]	Omicron [42]

**Table 3.** Parameters of the model described in figure 1 and equation (3.1). The probability with which masking causes infections to be mild (m) is unknown. Our default value is chosen to be substantial so that for illustrative graphs constructed with fixed m (figures 5, 6 in §5) the effect of mask-induced variolation is non-negligible. The recovery rates can be interpreted as the rates of ‘recovery or death’ since we do not explicitly model disease-induced mortality (cf. final paragraph of §3). The death rate *μ* refers to mortality from causes other than the focal disease. Note that mild illness is assumed to be associated with mild infectiousness. All birth rates were estimated for the years 2015–2020. We use a default latent period of *T*_lat_ = 3.7 days for all variants. The generation interval for an SEIR model is *T*_lat_ + *T*_inf_ [49, eqn (4.1)]. Setting *γ*_*x*_ = 1/*T*_gen,*x*_ in our model yields dynamics more similar to an SEIR version (cf. [49,52]), so it is a better approximation of the real world than an SIR version with 1/*γ* taken to be the observed mean infectious period. The transmission rate for severe infections ($\beta_{s}$) is set for each variant using equation (4.1) with m=0 and the associated $R_{0}$ estimate listed in table 2. After specifying $\beta_{s}$, we then set $\beta_{m}=(\beta_{m}/\beta_{s})\times \beta_{s}$.

parameter	meaning	expression or default value
m	probability that an infected individual develops *mild* illness	0.6
$\beta_{m}$	transmission rate from mildly infectious individuals	equation (4.1)
$\beta_{s}$	transmission rate from severely infectious individuals	equation (4.1)
$\beta_{m}/\beta_{s}$	ratio of transmission rates	1/2
$\gamma_{m}$	recovery rate from mild infections	1/*T*_gen,m_
$\gamma_{s}$	recovery rate from severe infections	1/*T*_gen,s_
*ν*	*per capita* annual birth rate [53]	0.0105 (Canada)
		0.0115 (UK)
		0.012 (USA)
*μ*	*per capita* annual death rate	*ν*
*δ*	rate of decay of immunity	1/*T*_imm_

## 4. Analysis

### 4.1. Basic reproduction number ($R_{0}$)

The contribution to $R_{0}$ from infectors who are mildly infectious is the probability of mild infection (m), times the transmission rate from mildly infectious individuals ($\beta_{m}$), times the expected time that a mildly infectious individual is infectious ($1/(\gamma_{m}+\mu )$). There is a similar contribution from severely infectious individuals, hence

4.1 $$R_{0}=m\frac{\beta_{m}}{\gamma_{m}+\mu }+(1-m)\frac{\beta_{s}}{\gamma_{s}+\mu }.$$

A formal calculation, applying the method of [54] to equation (3.1), yields the same expression.

### 4.2. Dimensionless stage durations

The mean time spent in the mildly infectious state, as a fraction of the mean lifetime, is

4.2a $$\varepsilon_{m}=\frac{\mu }{\gamma_{m}+\mu }.$$

Similarly, for severe infections, we have

4.2b $$\varepsilon_{s}=\frac{\mu }{\gamma_{s}+\mu }.$$

The mean across both types of infections is

4.2c $$\varepsilon =m\;\varepsilon_{m}+(1-m)\varepsilon_{s}.$$

It is worth noting that

4.3 $$\varepsilon \leq \max\left\{\varepsilon_{m},\varepsilon_{s}\right\}<1.$$

The mean duration of immunity, as a fraction of the mean lifetime, is

4.4 $$\eta =\frac{\mu }{\delta }.$$

### 4.3. Equilibria and stability

The system described by equation (3.1) always has a disease free equilibrium (DFE), at which *S* = *N* and all other compartments are empty. In addition, if $R_{0}>1$ then there is an endemic equilibrium (EE) given by

4.5a $$\;\frac{\hat{S}}{N}=\frac{1}{R_{0}},$$

4.5b $$\;\frac{\hat{I_{m}}}{N}=m\;\varepsilon_{m}(1-\frac{1}{R_{0}})\left(\frac{\eta +1}{\eta +\varepsilon }\right)$$

4.5c $$\mathrm{and}\;\;\frac{\hat{I_{s}}}{N}=(1-m)\;\varepsilon_{s}\left(1-\frac{1}{R_{0}}\right)\left(\frac{\eta +1}{\eta +\varepsilon }\right).$$

In the limit of permanent immunity (*δ* → 0, *η* → ∞), the final factors in equations (4.5*b*,*c*) simplify to 1. Since ε<1, decay of immunity (*η* < ∞) necessarily increases equilibrium prevalence. Theorem 2 of [54] establishes that $R_{0}=1$ is the boundary between local stability and instability of the DFE. In fact, the model (3.1) is a special case of the class of SIR models with multiple parallel infectious stages considered by Korobeinikov [55]. Consequently, theorem 1 of [55] establishes that the DFE is globally asymptotically stable (GAS) if $R_{0}\leq 1$, and that the EE is GAS if $R_{0}>1$.

### 4.4. Initial growth rate (*r*)

The initial exponential growth rate of an epidemic beginning near the DFE of equation (3.1) is the largest eigenvalue of the Jacobian derivative of the vector field $\left(\dot{S},\dot{I_{m}},\dot{I_{s}}\right)$ at the DFE. This Jacobian has a first column (− *δ* − *μ*, 0, 0), so −(*δ* + *μ*) is an eigenvalue. The other two eigenvalues are determined by the submatrix

4.6 $$\left(\begin{array}{cc} m\beta_{m}-(\gamma_{m}+\mu ) & m\beta_{s}\\ (1-m)\beta_{m} & (1-m)\beta_{s}-(\gamma_{s}+\mu ). \end{array}\right)$$

If we write, for convenience,

4.7 $$\beta =m\beta_{m}+(1-m)\beta_{s},$$

then the larger of the two eigenvalues of (4.6) is

4.8 $$r=\frac{1}{2}\{\beta -(\gamma_{m}+\gamma_{s}+2\mu )+\sqrt{{(\beta +(\gamma_{m}-\gamma_{s}))}^{2}-4m\beta_{m}(\gamma_{m}-\gamma_{s})}\}.$$

Note that, as written, the discriminant in equation (4.8) is manifestly positive if $\gamma_{m}\leq \gamma_{s}$, but it can also be written^1^

4.9 $${(\beta -(\gamma_{m}-\gamma_{s}))}^{2}+4(1-m)\beta_{s}(\gamma_{m}-\gamma_{s}),$$

which is manifestly positive if $\gamma_{m}\geq \gamma_{s}$. In the limit of equal mild and severe infectious periods, the initial growth rate is simply

4.10 $$r=\beta -(\gamma +\mu ),\;\text{if }\;\gamma_{m}=\gamma_{s}\equiv \gamma .$$

For any initial growth rate *r* (equation (4.8)), the *initial doubling time*, i.e. the time required for prevalence to double during the exponential growth phase, is

4.11 $$T_{2}=\frac{\log2}{r}.$$

### 4.5. Final size

If there is no source of new susceptibles (*ν* = *μ* = *δ* = 0), we can derive a final size relation based on equation (3.1). Following [56,57], we look for a constant of the motion that is the sum of log(*S*/*N*) and a linear combination of the other state variables. Solving for coefficients that cause the time-derivative of this expression to vanish, we find that

F(t)=log⁡SN−(1−m)(βmγm−βsγs)ImN+m(βmγm−βsγs)IsN  +(mβmγm+(1−m)βsγs)Rm+RsN

is a constant of the motion. In particular, *F*(0) = *F*(∞); consequently, since $\left(S,I_{m},I_{s})\to (N,0,0\right)$ as *t* → 0 and $\left(I_{m},I_{s})\to (0,0\right)$ as *t* → ∞ (and noting that the coefficient of the final term in equation (4.12) is $R_{0}$) we have

4.13 $$0=\log\frac{S(\infty )}{N}+R_{0}(\frac{R_{m}(\infty )+R_{s}(\infty )}{N}).$$

Writing *Z* = 1 − *S*(∞)/*N* and noting that

4.14 $$\frac{R_{m}(\infty )+R_{s}(\infty )}{N}=Z,$$

we obtain the standard final size relation [34,57],

4.15 $$\log\left(1-Z\right)=R_{0}Z.$$

With the same approach, but insisting that the coefficient of $R_{s}$ vanishes, we find another constant of the motion

4.16 $$F_{m}(t)=\log\frac{S}{N}+\frac{1-m}{m}\frac{\beta_{s}}{\gamma_{s}}\frac{I_{m}}{N}-\frac{\beta_{s}}{\gamma_{s}}\frac{I_{s}}{N}+\frac{1}{m}R_{0}\frac{R_{m}}{N}.$$

Considering the limits *t* → 0 and *t* → ∞ yields

4.17 $$\frac{R_{m}(\infty )}{N}=\frac{m}{R_{0}}\log\left(1-Z\right).$$

From this expression and equations (4.14) and (4.15), we therefore have exact expressions for the mild and severe attack rates,

4.18 $$Z_{m}\equiv \frac{R_{m}(\infty )}{N}=mZ\;\mathrm{and}\;Z_{s}\equiv \frac{R_{s}(\infty )}{N}=(1-m)Z.$$

Solving equation (4.15) for *Z* [57], we can write

4.19 $$Z_{s}(m)=(1-m)\left(1+\frac{1}{R_{0}(m)}W_{0}[-R_{0}(m)\;e^{-R_{0}(m)}]\right),$$

where *W*_0_ is the principal branch of Lambert’s *W* function [58]. In equation (4.19), we have emphasized the dependence of $Z_{s}$ on m (including the dependence of $R_{0}$ on m, equation (4.1)), since we are especially interested in understanding how an increase in the *proportion* of cases that are mild influences the expected *number* of severe infections.

### 4.6. Peak prevalence

Another way of writing equation (4.12) is

4.20 $$\frac{1}{R_{0}}\left(\frac{\beta_{m}}{\gamma_{m}}\frac{I_{m}}{N}+\frac{\beta_{s}}{\gamma_{s}}\frac{I_{s}}{N}\right)=\left(1-\frac{S}{N}\right)+\frac{1}{R_{0}}\log\frac{S}{N},$$

which does not depend explicitly on the probability m that an infection is mild (there is implicit dependence on m through $R_{0}$; equation (4.1)). In the limit that mild and severe infections have the same reproduction number ($\beta_{m}/\gamma_{m}=\beta_{s}/\gamma_{s}$), the left-hand side of equation (4.20) reduces to the total prevalence,

4.21 $$\frac{I}{N}=\frac{I_{m}+I_{s}}{N},$$

and equation (4.20) agrees exactly with the formula for the phase portrait *I*(*S*) of the standard SIR model. The right-hand side of equation (4.20) is maximized at $S=N/R_{0}$, so inserting $S=N/R_{0}$ into the equation (4.20) yields the peak prevalence formula for the standard SIR model,

4.22 $$\frac{I_{\mathrm{peak}}}{N}=1-\frac{1}{R_{0}}(1+\log R_{0}).$$

**Figure 3.**
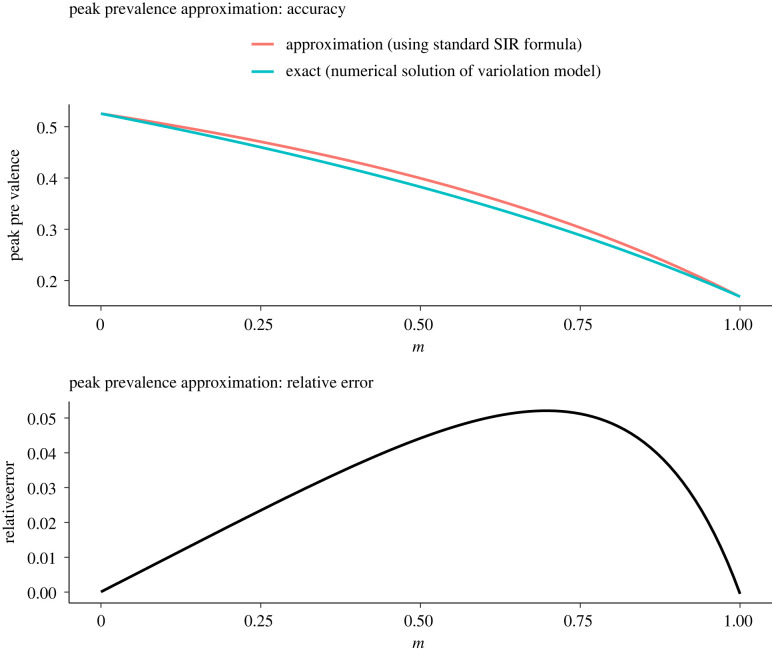
Accuracy and relative error of the peak total prevalence approximated using the formula for the basic SIR model (equation (4.22)), as a function of the probability that an infection is mild (m). Parameters other than m are set to the values specified in tables 2 and 3. For each m, the value of $R_{0}$ is computed using equation (4.1). The relative error is $\frac{SIR-TRUE}{TRUE}$, where TRUE refers to the peak total prevalence obtained by numerically solving equation (3.1), and SIR refers to equation (4.22). The maximum relative error of the approximation is 5.5%, for m=0.7 ($R_{0}=3.12$). In the extreme of perfect variolation (m=1), $R_{0}=R_{0,m}=2.02$, whereas in the extreme of completely ineffective variolation (m=0), $R_{0}=R_{0,s}=5.69$.

This formula will provide a good approximation to the peak total prevalence in our model (equation (3.1)) to the extent that the left-hand side of equation (4.20) approximates total prevalence near its peak. Figure 3 compares total prevalence (equation (4.21)) with the left-hand side of equation (4.20) for the full range of possible mask-induced increase in the probability of mild infection (0≤m≤1) with other parameters fixed at the values specified in tables 2 and 3. The figure indicates that approximating peak prevalence with the left-hand side of equation (4.20) is reasonable (the maximum relative error is 5.5%).

If we approximate the susceptible population *S* at peak total prevalence by $N/R_{0}$, then inserting $S=N/R_{0}$ into equation (4.20) provides an estimate of how prevalence at its peak is partitioned between mild and severe cases. Of course, we can approximate the value of *S* at peak total prevalence to any desired accuracy by solving equation (3.1) numerically; with that approach, we can use equation (4.20) to determine, to any desired accuracy, the partitioning of peak prevalence between mild and severe cases.

In practice, the peak prevalence of severe cases is what is most important from the point of view of stress on health care systems, and it is simplest to find the peak of $I_{s}$ directly from numerical solutions of equation (3.1). However, it is important to bear in mind that $I_{s}$ is the number of individuals who are still contributing to transmission dynamics but are suffering from disease that is so severe that they *will* need substantial healthcare. $I_{s}$ does not include people who have already been isolated in hospitals or other settings (and can therefore be considered unable to cause further infections). Thus, $I_{s}$ is a measure of upcoming healthcare *demand*, as opposed to the current *burden* on the system. The peak healthcare burden can be estimated roughly by multiplying the peak of $I_{s}$ by *T*_H_/*T*_gen,s_, where *T*_H_ is the mean length of stay in hospital and $T_{\mathrm{gen},s}=1/\gamma_{s}$ is the mean time spent in the $I_{s}$ compartment of our model (cf. tables 2 and 3).

## 5. Illustration of results for COVID-19

**Figure 4.**
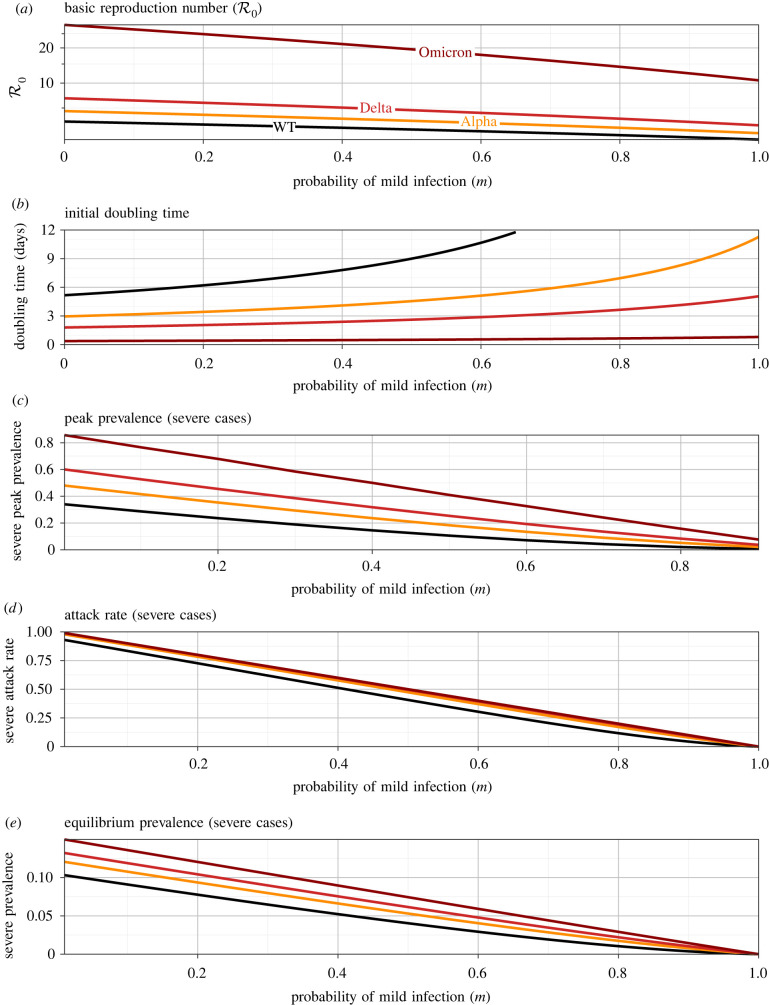
Epidemiological risk metrics as a function of the probability, m, that an infection is mild (as a result of mask-induced variolation). In each panel, results are shown for three situations, corresponding to the original SARS-CoV-2 wild-type, and the Alpha, Delta and Omicron variants (for which $R_{0}=3,4.5,6.75\;\mathrm{and}\;28.4$, respectively; cf. table 2). (*a*) Basic reproduction number (equation (4.1)); the estimated values of $R_{0}$ for each variant are assumed to be associated with ineffective variolation (m=0). All other parameters are set to the default values indicated in table 3 (the Canadian birth rate is assumed). (*b*) Doubling time *T*_2_ (equation (4.11)); for the original wild-type (WT), the curve extends far above the vertical maximum of the plotted graph (*T*_2_ → 65 days as m→1). (*c*) Peak prevalence of severe cases (§4.6). (*d*) Expected final proportion of the population that will have experienced a severe illness (equation (4.19)). (*e*) Equilibrium prevalence of severe cases (equation (4.5*c*)).

Figure 4 shows how several important epidemiological risk metrics depend on the variolating effect of masking, if it acts principally by increasing the probability of mild infection (m). Each panel shows three curves, associated with the estimated basic reproduction number for the original wild-type SARS-CoV-2 virus (WT, $R_{0}∼3$; [38]), the Alpha variant that began to spread in late 2020 ($R_{0}∼4.5$; [51]), the Delta variant that emerged in the spring of 2021 ($R_{0}∼6.75$; [39–41]) and the Omicron variant that emerged in late 2021 ($R_{0}∼28$; [42]). $R_{0}$ for each variant is assumed to take the observed value in the limit of no variolating effect (m=0) and then to decrease according to equation (4.1). All other parameters are fixed at the default values listed in tables 2 and 3; in particular, we assume that the transmission rates of mild and severe infections ($\beta_{m}$, $\beta_{s}$) are independent of their probability of occurrence (m, 1−m).

Figure 4*b* shows that the initial growth rate of the epidemic is strongly dependent on the probability m, and is more sensitive to m if the variant is more transmissible. As m is increased from 0 to 1, the initial doubling time increases from 5.1 to 65 days for WT (upper limit not shown on the graph), 2.9 to 11 days for Alpha, 1.8 to 5.0 days for Delta and 0.37 to 0.79 days for Omicron. (Note that these are estimates of expected doubling times in a completely susceptible population, whereas the doubling times actually observed when the later variants emerged were in populations that already had substantial levels of immunity from previous infections and vaccination.)

Figure 4*c*–*e* shows that risk measures related to severe infections decline substantially with m, as expected since severe illness is completely eliminated in the limit of perfect variolation (m→1; all cases mild). Variolation has relatively greater effect (on prevalence of severe illness) for more transmissible variants.

**Figure 5.**
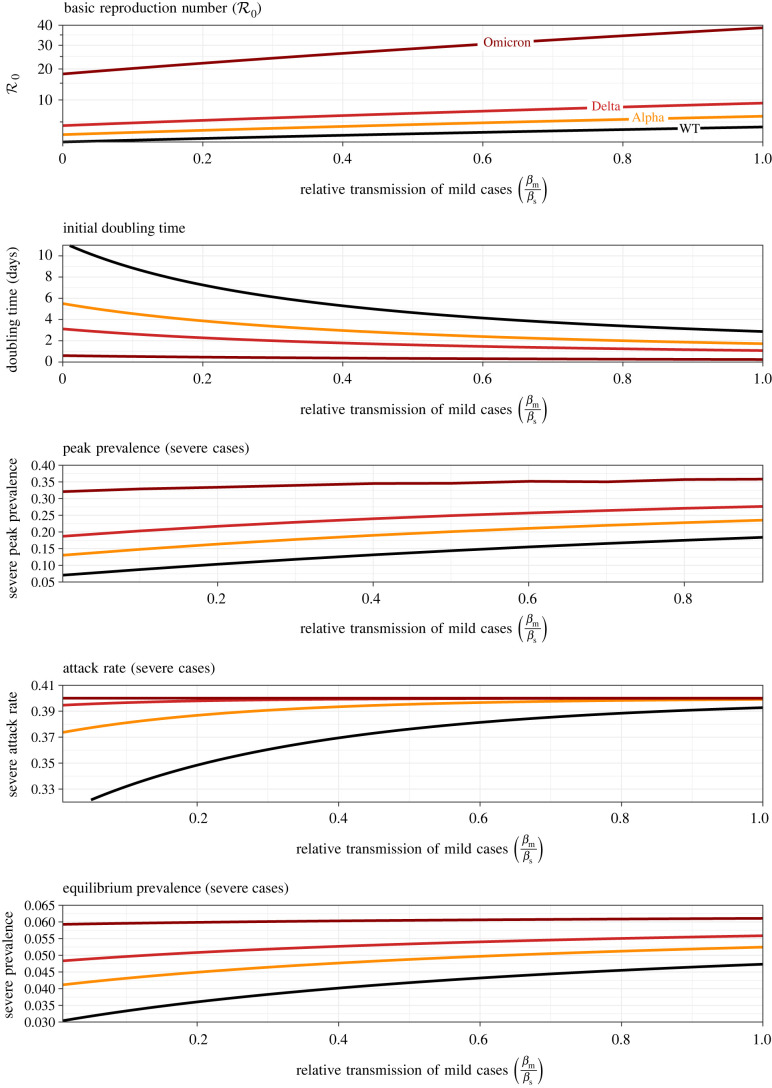
Epidemiological risk metrics as a function of the relative transmissibility of mild infections ($\beta_{m}/\beta_{s}$). The panels correspond to those in figure 4, but the vertical axis ranges are different. The graphs were generated by varying $\beta_{m}$ while fixing all other parameters at their default values. The estimated values of $R_{0}$ (listed in table 2) are associated with $\beta_{m}/\beta_{s}=1/2$ (the default ratio listed in table 3).

**Figure 6.**
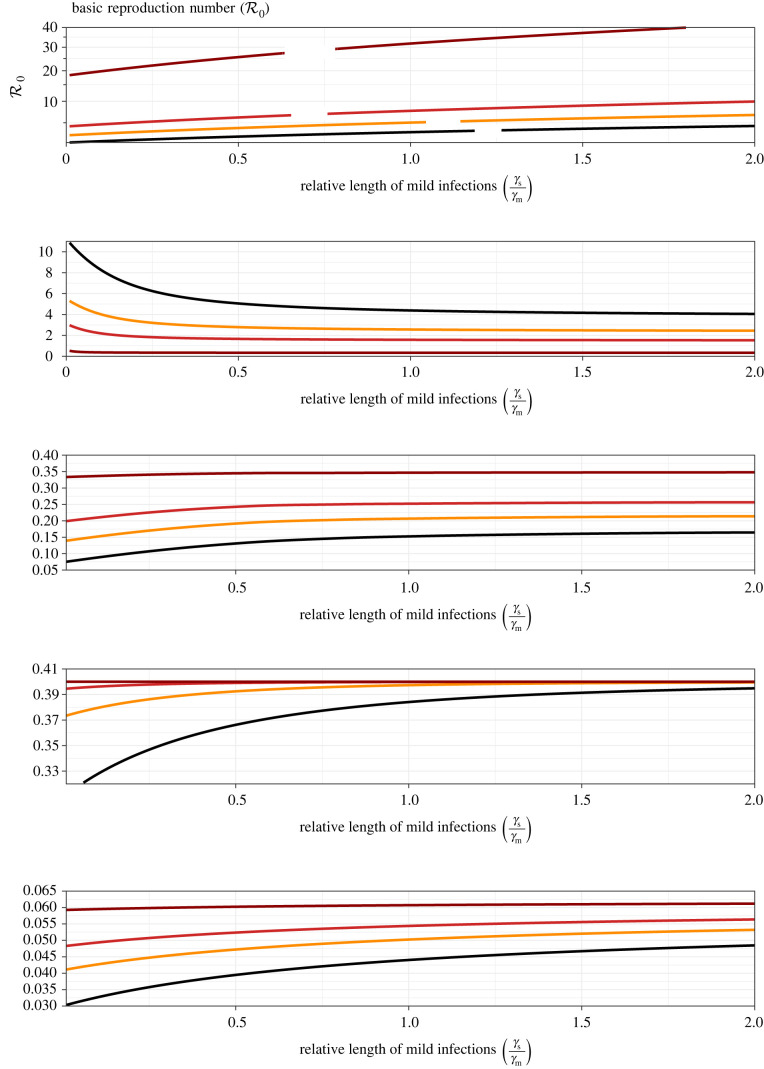
Epidemiological risk metrics as a function of the relative length of mild infections ($\gamma_{s}/\gamma_{m}$). The panels correspond to those in figures 4 and 5, but with different vertical axis ranges. The graphs were generated by varying $\gamma_{m}$ while fixing all other parameters at their default values. The estimated values of $R_{0}$ (listed in table 2) are associated with the ratio of generation intervals listed in table 3 ($\gamma_{s}/\gamma_{m}=T_{\mathrm{gen},m}/T_{\mathrm{gen},s}=(3.3+9)/(3.3+14)=0.71$).

Figures 5 and 6 show how the same risk metrics depend on the relative transmissibility of mild infections ($\beta_{m}/\beta_{s}$) and the relative length of mild infections ($\gamma_{s}/\gamma_{m}$). The horizontal scale in figure 5 ends at 1 because it is implausible that mild infections are intrinsically more transmissible than severe infections. By contrast, the horizontal scale in figure 6 extends beyond 1 because it is plausible that the time during which an infection can be transmitted is shorter for severe infections (e.g. severe cases are likely to be isolated sooner and more stringently). The similarities between figures 5 and 6 can be attributed to the fact that increasing $\beta_{m}$ or decreasing $\gamma_{m}$ has a similar effect on $R_{0}$ (equation (4.1)).

## 6. Discussion

We have explored the potential role of mask-induced variolation in reducing the impact of COVID-19 (or other directly transmitted infectious diseases). Our approach has been to analyse a highly idealized mathematical model (figure 1 and equation (3.1)) that captures the key mechanisms that we wished to investigate, namely the potential for masking to cause a proportion of infections to be mild, reduce the probability of infection upon contact and/or change the infectious period. Because our model does not attempt to be realistic in detail, conclusions we draw are qualitative only. However, the simplicity of the model has made it possible to derive results analytically, so qualitative conclusions can easily be drawn for pathogens with reproduction numbers or generation intervals that are different from those of SARS-CoV-2.

### 6.1. Summary of results

Our main results are analytical formulae that show how the model parameters—including the probability of mask-induced mild infection (m), the relative transmissibility of mild infections ($\beta_{m}/\beta_{s}$) and the relative length of mild infections ($\gamma_{s}/\gamma_{m}$)—influence the initial epidemic doubling time (equation (4.11)), the peak prevalence of severe infections (§4.6), the attack rate for severe infections (equation (4.19)) and the equilibrium prevalence of severe infections (equation (4.5*c*)). These results are illustrated for parameters that are representative of four variants of SARS-CoV-2 in figures 4–6.

— Figure 4 shows that if masking primarily influences the probability that an infection is mild (m), then more effective masking strongly affects transmission (reducing $R_{0}$) and lengthens the doubling time substantially, especially for less transmissible variants. In addition, the peak prevalence of severe cases is strongly affected (and the effect on peak prevalence is substantially greater for more transmissible variants). The expected number of severe cases during the initial wave of infections is also strongly dependent on m, but is not sensitive to transmissibility over the range of $R_{0}$ observed for SARS-CoV-2 variants. The equilibrium prevalence of severe cases also declines with m (equations (4.1) and (4.5*c*)).
— Figures 5 and 6 show the effects of greater transmissibility of mild infections (increasing $\beta_{m}$), and longer infectious periods of mild cases (increasing $1/\gamma_{m}$), respectively. The effects are similar, as might be expected given that $R_{0}∼\beta /\gamma$ (equation (4.1)).

Overall, increasing the effectiveness of mask-induced variolation—whether by increasing the probability that an infection will be mild, reducing the transmissibility of mild infections or reducing the length of mild infections—has the potential to drastically impact disease control, by slowing spread and reducing the magnitude of the epidemic peak (‘flattening the curve’ [59,60]), reducing the number of severe cases in the initial wave and reducing the prevalence of severe cases at equilibrium.

### 6.2. Limitations

The principal limitation of our analysis is that the hypothetical variolating effect of facemasks is unproven. We do not know that masking does tend to induce milder infections nor, if so, that variolated individuals attain a similar level of immunity as those who are infected in the absence of masks. Comments [61] and responses [62–64] to Gandhi & Rutherford’s initial article [17] make clear that further experimental and observational research is required. It is also worth noting that even if mask-induced variolation were very effective, promoting it could ‘implicitly encourage reckless behaviour’ [63] and consequently could present additional challenges for public health messaging.

### 6.3. Conclusion

Beyond qualitative conclusions, we are not likely to be able to make more powerful inferences without experimental studies that convincingly quantify the magnitudes of the effects that induce variolation from masking. If such experimental data do become available—and support the hypothesis that masking induces a substantial variolating effect—it will then be worth expanding our simple model (3.1) to include explicit latent periods, hospitalization, age and social structure (e.g. schools, workplaces) and heterogeneities in adherence to masking and other control measures. With appropriate data and more realistic models, we may be able to make quantitative inferences that could usefully inform policy decisions.

In the context of the highly transmissible Delta and Omicron variants [39–42], and the potential evolution of new SARS-CoV-2 variants that are even more transmissible and/or more successfully evade existing vaccines [65], a better understanding of the effectiveness of masking in promoting variolation could be of great value. At the time of writing, vaccines for children under 5 are not yet approved [66,67], but approval is expected soon [68,69]. While vaccine availability for people of all ages is imperative, substantial vaccine hesitancy [70] and breakthrough infections among the vaccinated [71–74], make achieving herd immunity through vaccination an unattainable target at present. If that situation persists, potential mask-induced variolation could contribute to COVID-19 control as we transition to endemicity.

## Data Availability

This article has no additional data.

## Endnote

1 The equivalence of the expressions for the discriminant in (4.8) and (4.9) can be verified directly, or by recognizing that the results must be invariant to changing the labelling of mild and severe: *m* → *s*, *s* → *m*, *m* → (1 − *m*).
